# Comparing Neural Correlates of Human Emotions across Multiple Stimulus Presentation Paradigms

**DOI:** 10.3390/brainsci11060696

**Published:** 2021-05-25

**Authors:** Naveen Masood, Humera Farooq

**Affiliations:** 1 Electrical Engineering Department, Bahria University, Karachi 75260, Pakistan; 2 Computer Science Department, Bahria University, Karachi 44000, Pakistan; humerafarooq.bukc@bahria.edu.pk

**Keywords:** classification, common spatial pattern (CSP), electroencephalography (EEG), emotional imagery, emotions, feature extraction

## Abstract

Most electroencephalography (EEG)-based emotion recognition systems rely on a single stimulus to evoke emotions. These systems make use of videos, sounds, and images as stimuli. Few studies have been found for self-induced emotions. The question “if different stimulus presentation paradigms for same emotion, produce any subject and stimulus independent neural correlates” remains unanswered. Furthermore, we found that there are publicly available datasets that are used in a large number of studies targeting EEG-based human emotional state recognition. Since one of the major concerns and contributions of this work is towards classifying emotions while subjects experience different stimulus-presentation paradigms, we need to perform new experiments. This paper presents a novel experimental study that recorded EEG data for three different human emotional states evoked with four different stimuli presentation paradigms. Fear, neutral, and joy have been considered as three emotional states. In this work, features were extracted with common spatial pattern (CSP) from recorded EEG data and classified through linear discriminant analysis (LDA). The considered emotion-evoking paradigms included emotional imagery, pictures, sounds, and audio–video movie clips. Experiments were conducted with twenty-five participants. Classification performance in different paradigms was evaluated, considering different spectral bands. With a few exceptions, all paradigms showed the best emotion recognition for higher frequency spectral ranges. Interestingly, joy emotions were classified more strongly as compared to fear. The average neural patterns for fear vs. joy emotional states are presented with topographical maps based on spatial filters obtained with CSP for averaged band power changes for all four paradigms. With respect to the spectral bands, beta and alpha oscillation responses produced the highest number of significant results for the paradigms under consideration. With respect to brain region, the frontal lobe produced the most significant results irrespective of paradigms and spectral bands. The temporal site also played an effective role in generating statistically significant findings. To the best of our knowledge, no study has been conducted for EEG emotion recognition while considering four different stimuli paradigms. This work provides a good contribution towards designing EEG-based system for human emotion recognition that could work effectively in different real-time scenarios.

## 1. Introduction

Emotional state recognition plays an important role in the research area of human–computer interaction. The ability to identify a person’s emotional state based on relatively easily acquired scalp electroencephalographic (EEG) data could be of clinical importance for anger management, depression, anxiety, or stress reduction, especially for persons with communication disabilities. Human emotion refers to a complex psychological state comprising three components, i.e., user experience, physiological response, and behavioral and expressive reactions [1,2,3]. Different categories of emotional states are identified as fear, disgust, pride, happiness, anger, etc. [4,5,6,7,8,9,10,11,12]. Various studies have been conducted to find how the EEG signals correlate to human emotions [13,14,15].While reviewing the literature, we found that most of the EEG emotion recognition-based studies have used a single method to elicit emotions [13,14,15,16,17,18,19,20,21,22,23,24,25,26,27,28,29,30,31,32,33,34,35,36,37,38,39,40,41,42,43,44,45,46,47]. Thus, the question as to “whether different stimuli of elicitation for same emotion generate any subject and stimuli independent correlates” remains unanswered. Furthermore, concerning the domain of human emotion recognition, we found that there are publicly available datasets such as DEAP, SEED, etc., that have been used in a large number of studies targeting EEG-based human emotional states recognition [21,22,23,24]. However, because one of the major concerns and contributions of this work is the classification of emotions while subjects are experiencing different stimuli-presentation paradigms, we needed to perform new experiments. While researching studies of EEG classification for different mental activities, we found common spatial patterns (CSP) to be one widely used approach specially for motor imagery classification. CSP has also been used for the classification of different stress or workload levels while subjects are performing different activities. Within the domain of emotion recognition, few researchers have used CSP for this purpose [15].

In the presented work, a multi-modal emotion elicitation paradigm is proposed to investigate whether the same signatures of emotions exist if induced by different methods of elicitation. This study is focused on recording and analyzing EEG data of human subjects for examining differences between joy, fear, and neutral emotions, using different methods of stimulus for elicitation. Along with neutral brain state, we attempted to explore the EEG correlates of joy and fear as positive and negative emotions, respectively. This work is an extension of our previous work [48],in which we have conducted experiments with dual stimuli emotion-presentation paradigms specifically for fear. In this study, we have enhanced the experiment and extended the dataset for three emotional states, fear, neutral, and joy, to achieve the target for emotion recognition. To the best of our knowledge, no study has been conducted focusing on distinguishing different emotional states with four different stimulus-presentation paradigms and finding common neural correlates, independent of subjects and stimuli.

The considered paradigms include emotional imagery, pictures, sounds, and audio–video movie clips. In cases of memory recall/self-induced/emotional imagery, we are primarily dealing with inwardly visualized, imagined, or felt emotions evoked by the subject’s own imagination or the recall of emotionally loaded memories [8]. The participants are requested to become involved or immerse themselves in the prolonged, self-paced recall of emotion imagination, usually with closed eyes. Moreover, in this study, audio–video clips were shown to the subjects to elicit emotions. Furthermore, pictures from the IAPS [49] database and sounds from IADS [50] have also been used to evoke emotional states in the participants. For data analysis, each paradigm in each dataset has been analyzed separately. From the recorded data, bandpass EEG features were extracted, and spatial filters were obtained based on the common spatial pattern (CSP) algorithm. In order to perform spectral analysis, different setups for frequency bands were chosen and classification performance was compared in each of the selected bands. The main contributions of this work are mentioned as follows:

- We have presented a novel dataset that records EEG data for fear, neutral, and joy human emotional states evoked with four different stimuli presentation paradigms;
- Identification of the most relevant spectral bands and brain regions with respect to each paradigm;
- CSP has been widely used in different scenarios of EEG-based BCI applications such as motor imagery; therefore, this work attempts to investigate if it is also a good choice for emotion recognition.

## 2. Related Work

While studying EEG-based emotion recognition-based studies, the reader may find two directions: analyzing frequencies to either learn about emotion with respect to cortical activations/ERP response, or utilizing frequencies to classify emotion from the field of computer science [31,33,34,35,39,40,51]. In next sub-sections, we will cover these two aspects.

### 2.1. Cortical Activity Perspective

While performing EEG spectral analysis, the signal can be analyzed in different spectral bands: delta, theta, alpha, beta, and gamma. Each of the spectral bands are studied by EEG community to analyze and investigate different human emotional states. We will discuss each one of them in the following sub-sections. Moreover, Table 1 summarizes the findings observed in emotion recognition studies based on EEG data.

#### 2.1.1. Delta Band

In a study conducted by Knyazev et al. [36], EEG delta oscillation responses for different emotional states that included angry, happiness, and neutral conditions were investigated. They found stronger delta synchronization for angry and happiness states as compared to neutral. Similarly, Güntekin and Başar [29] performed a study on emotion recognition and conducted gender-wise comparison. They found that female participants showed higher delta oscillation responses as compared to males.

#### 2.1.2. Theta Band

Various EEG studies have been conducted to examine theta oscillation responses for different emotional states evoked with different stimulus presentation paradigms [33,34,35]. Alberto Ara and Marco-Pallares studied frontal theta power synchronization for music-evoked pleasantness. EEG was recorded with 25 participants. Stronger theta oscillation synchronizations were reported in temporal and frontal sites with higher degrees of pleasantness [33]. In a study conducted by Balconi and Lucchiari [34], stronger frontal theta synchronization was observed for emotional stimuli as compared to neutral stimuli. Zhang et al. [35] also found an increase in theta synchronization for frightening facial stimuli in comparison to a neutral stimulus.

#### 2.1.3. Alpha Band

Performing the literature review, we found various studies which analyzed alpha oscillation responses while the participants were experiencing different emotional states [26,27,28,29,30,31,32]. Often, within this domain, asymmetric frontal cortical activity is studied by comparing alpha oscillation responses across the left and right brain regions. Lee et al. [26] reported a decrease in alpha power within the right and left frontal brain regions. Otten and Jonas [52] conducted a study to evoke emotions using emotion-evoking words, and compared pleasant, unpleasant, and neutral conditions. Meng et al. [27] and Mennella et al. [28] also studied alpha oscillations for emotions evoked by displaying pictures from the International Affective Picture System (IAPS) database. They found a decrease in alpha power post-stimulus presentation. However, some other studies found contrasting results and reported increases in alpha oscillation responses for different emotional states [29,30,31].

#### 2.1.4. Beta Band

The relationship for beta oscillation responses with EEG data recorded for different emotional states has been reported in different studies [29,32,37,38]. In their study, Holler et al. [32] recorded EEG data while participants listened to their favorite music. The study reported beta cortical activity while listening to music in most of the subjects. Schubring and Schupp [53] conducted a study with sixteen participants to analyze EEG-based emotional data while the participants were viewing erotic and romantic pictures. The study reported a decrease in alpha and beta oscillation power responses in posterior and anterior sites for erotic images in comparison to romantic images. Schutter, Putman, Hermans, and van Honk [38] observed a strong response in beta oscillations at the parietal region while showing angry faces to the participants. Güntekin and Başar [29] also reported a stronger beta response at frontal and central electrodes for angry faces in comparison to happy faces. Moreover, Güntekin and Başar [37] found a higher beta response for negative emotions in comparison to positive emotions while subjects were viewing IAPS images.

#### 2.1.5. Gamma Band

Gamma oscillation responses have also been analyzed by researchers in different EEG- and MEG-based studies [25,54,55,56]. Eijlers et al. [25] reported lower gamma oscillation responses at temporal and frontal regions for happy emotions. Sato et al. [54] reported stronger gamma oscillation responses in the case of negative emotions as compared to neutral and happy states. Jung et al. [55] recorded EEG data for different emotions that included happiness, fear, disgust, neutral, and angry. Their study reported higher gamma oscillation responses for negative states at the lateral orbitofrontal brain region.

### 2.2. Classification Performance Perspective

With respect to classification performance perspective, here again we observed variations in findings and results amongst different studies targeting emotion recognition for different spectral bands. In their research, Li and Lu [39,40] concluded that gamma frequency band plays major role in emotion recognition. Zhang et al. [41], Jatupaiboon et al. [42], and Zheng et al. [43] also found that higher frequency bands provide a more significant contribution to emotion classification as compared to lower frequency ranges. In contrast to these studies, Shahabi et al. [44] found better classification accuracies in theta, alpha, and beta bands. Eijlers et al. [25] reported a strong classification for happy and disgust emotions in higher frequency ranges. In the case of negative emotions, including sadness and fear, strong differences were found for the alpha frequency band at the centroposterior region.

While reviewing the literature, we found that most of the EEG emotion recognition-based studies have used a single method to elicit emotions [25,26,27,28,29,30,31,32,33,34,35,36,37,38,39,40,41,42,43,44]. Thus, the question as to “whether different stimuli of elicitation for same emotion generate any subject and stimuli independent correlates” remains unanswered. Furthermore, concerning the domain of human emotion recognition, we found that there are publicly available datasets such as DEAP, SEED, etc., that have been used in a large number of studies targeting EEG-based human emotional state recognition. However, because one of the major concerns and contributions of this work is the classification of emotions while subjects experience different stimuli-presentation paradigms, we needed to perform new experiments. In the presented work, a multi-modal emotion elicitation paradigm is proposed to investigate whether the same signatures of emotions exist if induced with different methods of elicitation.

Ethical approval for the study was obtained from the Local Ethics Committee of Bahria University, Pakistan (approval code: ERC/ES/001).

## 3. Methodology

The proposed methodology comprises different phases. The first, in Section 3.1, concerns EEG data acquisition and includes an explanation of the experimental setup and protocol along with details of the participants and EEG device used for recording data. The next Section 3.2 explains the data analysis mechanism for EEG data acquired. In order to perform data analysis, we analyzed recorded EEG data using MATLAB toolboxes EEGLAB [57], Emotiv Xavier Test bench [58], and RCSP [59].

### 3.1. EEG Data Acquisition

We conducted experiments with twenty-seven young university students (14 female; 13 male). The mean age of the subjects was 21 years. The students were enrolled in Bachelor’s and Master’s of Computer Science programs. Before conducting the experiments, the purpose of study was explained, and consent forms were signed by all participants. Overall, thirty-five students filled in the form. Out of them, twenty-seven students gave their consent to participate in the experiments. The experimental study involving human participants described in this research work was approved by the local ethics committee of Bahria University, Pakistan. If any participant felt discomfort or for any other reason, they had the option to quit the experiment at any point. The data from two subjects (one male and one female) were excluded because they did not complete the experiments. An Emotiv EPOC EEG Headset was used for recording brain signals while subjects were performing the experiments. This is a wireless headset that requires less placement time and effort and offers improved mobility and flexibility as compared to other medical-grade EEG headsets [29,30,31,32,33]. The EEG signals were recorded with a sampling frequency of 256 Hz from the Emotiv EPOC headset with fourteen EEG channels, namely, AF3, AF4, F3, F4, FC5, FC6, F7, F8, T7, T8, P7, P8, O1 and O2. The data were recorded with the provided Emotiv software, named ‘Emotiv Xavier Test bench’. Fourteen (14) active electrodes on the headset are arranged according to the 10–20 international system.

The experiments were conducted in a laboratory environment. In the first stage, a briefing was presented to the subjects with respect to the purpose of the experiment being conducted. They were informed that EEG signals would be recorded while experiencing four different emotion-inducing paradigms. A questionnaire was given to each participant in which they wrote about any incident of their real life that was associated with fear or joy emotions. While performing the experiments, the participant was requested to stay still and relaxed. For stimuli presentation, an LCD screen was placed at a distance of about 50 cm from the participant. The following four scenarios/paradigms were conducted with each participant:

- Paradigm1—EI (Emotional Imagery/Self-Induced)
- Paradigm2—VI (Video-Induced)
- Paradigm3—SI (Sound-Induced)
- Paradigm4—PI (Picture-Induced)

For each paradigm, three emotional states of fear, neutral and joy were considered. For each emotional state in each paradigm, we conducted a different number of trials with each participant, as mentioned in Table 2. For emotional imagery (EI) and video-induced (VI) paradigms, a total of thirty trials were conducted. In the case of picture- and sound-induced scenarios (PI and SI scenarios, respectively), each experiment consisted of 135trials. The time duration of each trial is also mentioned in the table. For the EI paradigm, a single trial consisted of 60 s. In the PI paradigm, the image or picture was shown for 7 s. In the case of video-induced (VI) stimulus, the video clips played were of different durations, ranging from 60 to 180 s. Sounds were played for 6 to 10 s in the case of the SI paradigm. An explanation of experiments for each paradigm is given in following sub-sections.

#### 3.1.1. Paradigm1—EI (Experiments Performed Based on Emotional Imagery)

The trial in this paradigm started with 5 s of baseline recording. Then, the participant was asked to recall the relevant memory for the considered emotional state specified in the questionnaire. The subject was verbally signaled by the researcher to start the activity, which continueduntil60 s. EEG recording was then stopped. The participant was given a 30 s break after each trial and asked to rate the arousal level and specify the emotional state experienced during the experiment. The sequence for a single trial was as follows:

1. Baseline signal collection for 5 s;
2. The activity is initiated after a verbal signal;
3. The incident/memory/imagination mentioned in the questionnaire is recalled by the participant;
4. The activity is stopped after 60 s;
5. A time of 30 s is provided to rate the arousal level and specify the emotional state.

#### 3.1.2. Paradigm2—VI (Experiments Performed While Viewing Videos)

In this paradigm, different videos were shown to the participants in each trial. The list of videos shown to the participants is presented in Appendix A, Table A1. A total of thirty videos were shown alternatively with respect to fear, neutral, and joy emotions. The sequence for a single trial was as follows:

1. Baseline signal collection for 5 s;
2. Video clip is displayed after verbal signal. The activity is initiated;
3. Participant views the movie clip being displayed for 120–180 s;
4. The activity is stopped;
5. A time of 30 s is provided to rate the arousal level and specify the emotional state.

#### 3.1.3. Paradigm3—SI (Experiments Performed While Listening Sounds)

During each trial of this paradigm, the participants listened different sounds. Sounds were selected from the International Affective Digitized Sounds (IADS) database [50] consisting of 167 sound clips. The length of each clip consisted of 6 s. In total, 100 subjects provided ratings for valence, dominance, and arousal levels for each sound clip. The IADS database provides a good range of emotional states. Moreover, the stimuli in the IADS database have been excerpted from real-life events or scenarios. In order to minimize the possible variance of response from participants from different socio-cultural backgrounds, these scenarios were carefully selected while developing the IADS database [60]. For example, in order to induce positive pleasant emotions, the sounds of bird’s merry chirping, stream water flowing, or children’s laughter are used. In order to induce negative emotions for fear, sounds including a woman screaming, a woman crying, or a car crash are used. In our work, a total 135 sounds were played alternatively with respect to fear, neutral and joy. The sequence for a single trial is as follows:

1. Baseline signal collection for 5 s;
2. The activity is initiated after a verbal signal and the sound is played;
3. Sound continues for 5–10 s;
4. The activity is stopped;
5. A time of 30 s is provided to rate the arousal level and specify the emotional state.

#### 3.1.4. Paradigm4—PI (Experiments Performed While Viewing Pictures/Images)

During each trial of this paradigm, the participants viewed different images/pictures on the screen. Pictures were selected from the International Affective Picture System (IAPS) database [49]. IAPS was developed by the NIMH Center for Emotion and Attention at the University of Florida. It aims to provide standardized visual stimuli to the research community working in the domain of human emotional state analysis and recognition. The database consists of 700 colored images that have been collected over the span of ten years. The pictures and images in the database were selected, keeping in consideration broad range of arousal, valence, and dominance of human emotions along with minimizing the influence from different cultures, societies, and religions [61]. In our work, a total of forty-five pictures were shown with respect to each emotional state. The sequence for a single trial is as follows:

1. Baseline signal collection for 5 s;
2. The activity is initiated after a verbal signal;
3. The picture/image is displayed for 5–7 s;
4. Picture disappears;
5. A time of 30 s is provided to rate the arousal level and specify the emotional state.

**Figure 1:**
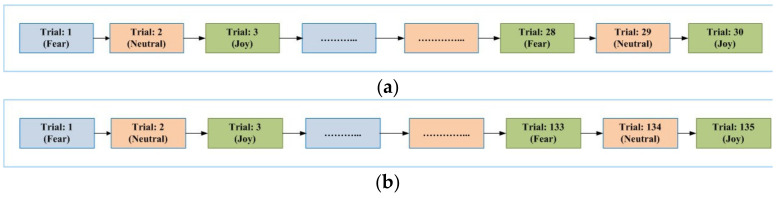
(**a**) Sequence of trials with respect to the emotional states in EI and VI paradigms. (**b**) Sequence of trials with respect to the emotional states in PI and SI paradigms.

**Figure 2:**
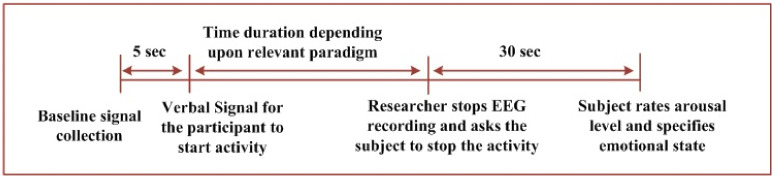
Block diagram of the experiment while conducting a single trial for any emotional states out of fear, joy, and neutral.

The general sequence diagram for the experiments in each of the considered paradigms is elaborated in Figure 1. The figure shows the sequence of trials for each emotional state in each of the considered paradigm. Figure 1a shows the sequence of trials for EI and VI scenarios. Figure 1b represents the sequences for PI and SI paradigms. Figure 2 provides general details of a single trial conducted. The next sub-section elaborates details regarding data analysis for the acquired signals from 64 EEG electrodes.

### 3.2. Data Analysis

**Figure 3:**
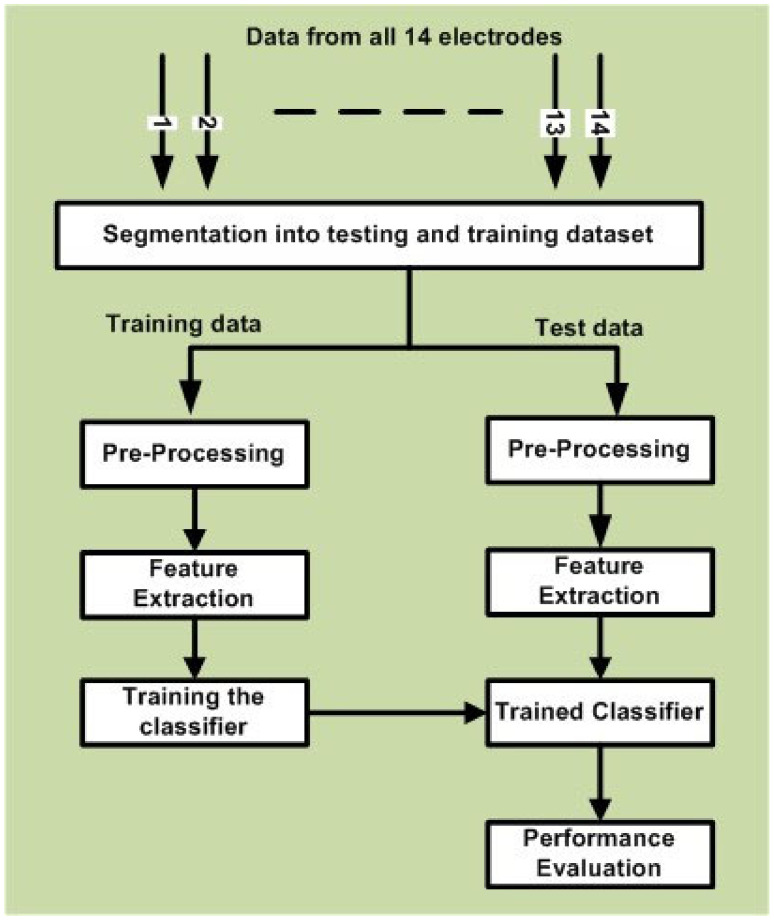
Block diagram explaining the data analysis process.

In this study, we performed data analysis by considering data for each stimulus paradigm separately. As mentioned earlier, this sub-section explains the data analysis mechanism for EEG data, as displayed in Figure 3. In order to perform data analysis, we analyzed the recorded EEG data using MATLAB toolboxes EEGLAB [57], Emotiv Xavier Test bench [58], and RCSP [59].

#### 3.2.1. Segmentation of Recorded EEG Data into Trials and Epochs

As mentioned in Figure 3, the first stage for data analysis is related to the ‘Segmentation of data into training and testing trials and epochs’. For each trial of any specific paradigm under consideration, features were extracted from the time duration mentioned in Table 2 For EI and VI paradigms, the last 55 s were considered for data analysis, while in the case of PI and SI scenarios, the last 5 s of each trial were considered. EEG data in each trial were divided into non-overlapping time windows of one second (1 s) long, which are referred to as an epoch. In this study, for emotional state classification, we worked with a 10× 10 cross-validation strategy in which the epochs were distributed into ten partitions. Out of ten, nine partitions (i.e., 90% of the data) were considered for training purposes, whereas the remaining one partition (i.e., 10% of the data) was considered for testing. The process was repeated for ten times with different dataset splits. Let us consider the case for VI paradigm. We had a total of 30 trials in this paradigm, as mentioned in the table. Now, for 10 × 10 cross-validation, each subset out of 10 contained3 trials. In each run for cross-validation, any one subset, i.e., 3 trials, were considered for testing purposes, and the remaining 9 subsets, i.e., 27 trials, were considered for training.

#### 3.2.2. Pre-Processing (Filtering, Artifact Removal and Epochs Rejection)

As mentioned earlier, each of the segmented trials from fourteen electrodes were recorded at a sampling rate of 256 Hz. Prior to data analysis, artifacts generated from ocular and muscle movements were removed using independent component analysis (ICA) and max–min-based approaches. In cases of a max–min approach, an epoch is rejected if it has amplitude difference greater than 150 μV between the maximum and the minimum amplitude values. The ICA algorithm is an advanced technique for artifact removal while working on EEG data. The algorithm has proven its capability for isolating neurally generated as well as artifactual sources residing in recorded EEG signals [40,57,61]. The EEGLAB function ‘runica’ [57] has been used in this work. As mentioned earlier, data analysis for each stimulus paradigm has been performed independently.

Now, the segmented pre-processed epochs are considered for baseline correction. As elaborated in the previous section, baseline recordings were performed for each paradigm for each trial. We considered −200 ms prior to the stimulus onset as the baseline interval. For this interval, the average amplitude value was subtracted from each trial after stimulus presentation. Then, the baseline-corrected EEG signals were bandpass-filtered in different frequencies bands as follows: delta (1–3 Hz), theta (4–7 Hz), alpha (8–13 Hz), beta (14–30 Hz), low gamma (31–50 Hz), and high gamma band (51–70 Hz). A Butterworth filter in the order of 5 was used. This operation produced six bandpass-filtered datasets for each of the subjects under consideration.

#### 3.2.3. Feature Extraction Based on CSP

Common spatial patterns (CSP) are a widely used algorithm for the classification of EEG motor imagery data. In this work, we used this algorithm for emotion recognition. The algorithm optimally differentiates two classes of EEG signals based on the simultaneous diagonalization of covariance matrices for each class [59]. In this work, three emotional states were considered. We had three scenarios: fear vs. joy; joy vs. neural; and fear vs. neutral. The baseline-corrected preprocessed EEG data in a single trial were represented as the matrix *XN × T*, where Nis the number of EEG electrodes and T is the number of time points recorded in each epoch of 1 s from each of the considered electrodes corresponding to two patterns (fear and joy). Here, the CSP algorithm was employed to obtain a projection matrix. The first and last m columns of the projection matrix were considered to construct a new matrix, *ω € XN × 2m*. In a conventional CSP approach, an input data matrix *XN × T* is generally transformed as:

(1) $$z=\omega^{T}\;X$$

The rows of ω and columns of *ω*-1 are termed as spatial filters and common spatial patterns, respectively. The spatial filters obtained from CSP are optimized separately for each frequency band such that it maximizes the variance of the projected signal for one class, whereas minimizing the other. The normalized spatial covariance matrix can be computed as:

(2) $$C=\frac{XX^{T}}{tr(XX^{T})}$$

where *X*^T^ refers to the transpose of EEG data matrix *X*, and *tr* represents the sum of diagonal elements of two emotional states under consideration. The CSP algorithm simultaneously diagonalizes the data matrices for both classes by designing *ω* such that it satisfies *ω*^T^*C*_1_*ω* = λ_1_ and *ω*^T^*C*_2_*ω* = λ_2_. Here, λ_1_ and λ_2_arediagonal matrices that satisfy λ_1_+ λ_2_ = *I.*

The CSP projection matrix is computed based on eigenvalue decomposition. A small number of signals, *m*, can effectively differentiate between the classes while training the classifier. The signal *Yq*(*q = 1 to 2m*) that maximizes discrimination is associated with the highest values for λ*1* and λ*2* [59,62]. Feature vectors *f_q_* are computed as follows:

(3) $$f_{q}=\log(\frac{var\;\left(Y_{q}\right)}{\sum_{i=1}^{2m}var\;\left(Y_{i}\right)})$$

In this study, log variances based on CSP were generated as features for the emotional states in consideration. Log-variance of bandpass-filtered signals relates to the signal power/power spectral density PSD in that corresponding frequency band for a given emotional state.

#### 3.2.4. Classification with Linear Discriminant Analysis

For classification purpose, this work considered linear discriminant analysis (LDA), assuming that the features extracted from CSP operation are based on different multivariate Gaussian distributions, with different known means, and a commonly known covariance matrix for each of the considered emotional state [63,64]. Based on estimates of the common covariance C and class-wise means *μ*_1_ and *μ*_2_, the weight vector *ω* of the classification function is determined by

(4) $$\;\omega =C^{-1}{\left(\mu_{2}-\mu_{1}\right)}^{}$$

The class-wise means are estimated as

(5) $$\mu =\frac{1}{n}\overset{n}{\underset{t=1}{\sum }}x_{i}$$

For multiple-class classification, researchers have worked with pair-wise strategies that include one-versus-one or one-versus-rest. Although LDA is basically designed for binary classification, using pair wise strategy the algorithm has performed quite successfully within the domain of EEG data classification [17,18,19,20]. In order to implement three-class emotional states classification using LDA, we adopted a one-versus-rest strategy, yielding three binary classifiers. More specifically, each binary classifier was trained considering the epochs from a given emotional state as positive labels and all other epochs from the remaining/rest of the emotional states as negative labels. Discrete classification was performed such that a class was assigned to each epoch. Let us consider the case for VI paradigm. In this case, features were calculated on a basis of 1 s epochs from the last 55 s of each trial, resulting in 55 × 10 = 550 epochs from the ten trials, as mentioned in Table 2. Each extracted epoch was then associated with a label y(t) є [+1, −1], as explained before. The sampling frequency was 256 Hz; therefore, each epoch was a matrix of 256 rows and 14 columns (due to them any electrodes). To perform classification, the trials were divided into the training set and testing set. CSP operation was then applied to compute bandpass features for both training and testing datasets. Each epoch x(t) was associated with the label y(t) ∈ [+1, −1] such that epochs from fear state were labeled as +1, while epochs for joy and neutral states were labeled as −1. Similarly, in cases of joy state classification, samples for joy were assigned a +1 label and the rest of the epochs from joy and neutral states as −1. As explained earlier, in order to perform emotional state classifications, we worked with a 10 × 10 cross-validation strategy in which the epochs were distributed into ten partitions. Out of ten, nine partitions (i.e., 90% of the data) were considered for training purpose, whereas the remaining one partition (i.e., 10% of the data) was considered for testing. The process was repeated ten times with different dataset splits. Let us consider the case for VI paradigm. We had total 30 trials in this paradigm, as mentioned in Table 2. For 10-fold cross-validation, each subset out of 10 contained3 trials. Thus, in each run for cross-validation, any one subset, i.e., 3 trials, were considered for testing data, and the remaining 9 subsets, i.e., 27 trials, were considered for training purpose.

### 3.3. Statistical Analysis

As mentioned in the previous section, band power features were calculated for each emotional state in each of the four considered paradigms. An average absolute power value for the following five regions: temporal T, frontal F, central C, parietal P, and occipital O, for each condition (fear and joy), was calculated separately for each frequency band. An average of the pre-experimental or baseline absolute power was used to determine the individual power during no emotional activation state. From this reference power value, individual power changes during stimuli presentation were determined as the relative stimulus-related change. Changes in band power can be defined as the percentage of decrease/increase in band power during a test interval during stimulus as compared to a reference interval before stimulus. For statistical analysis, used data from the percentage change in band power for each subject for each emotional state in all considered frequency bands and brain regions. The data were subjected to a repeated measures analysis of variance (ANOVA) with three repeated factors: emotional states (fear, joy), spectral band (delta, theta, alpha, beta, gamma), and region (temporal T, frontal F, central C, parietal P, and occipital O) for each stimulus (EI, PI, SI, and VI).

## 4. Experimental Results

### 4.1. Classification with Respect to Specific Stimulus Presentation Paradigm

In the presented work, along with neutral brain state, we attempted to explore the EEG correlates of joy and fear emotions evoked with four different emotion-evoking paradigms. As explained in the previous section, we performed data analysis by considering data for each stimuli presentation paradigm separately. It considers discrete classification of the samples such that a class is assigned to each sample. For each block of each of the considered emotion evoking paradigms, features were extracted from the relevant time segment. Ten-fold cross-validation was performed by splitting the dataset in ten equal parts. The features extracted from CSP were fed to the LDA classifier for evaluating classification performance in the different considered spectral bands of delta band (1–3 Hz), theta band (4–7 Hz), alpha band (8–13 Hz), beta band (14–30 Hz), and gamma band (31–50 Hz).

**Figure 4:**
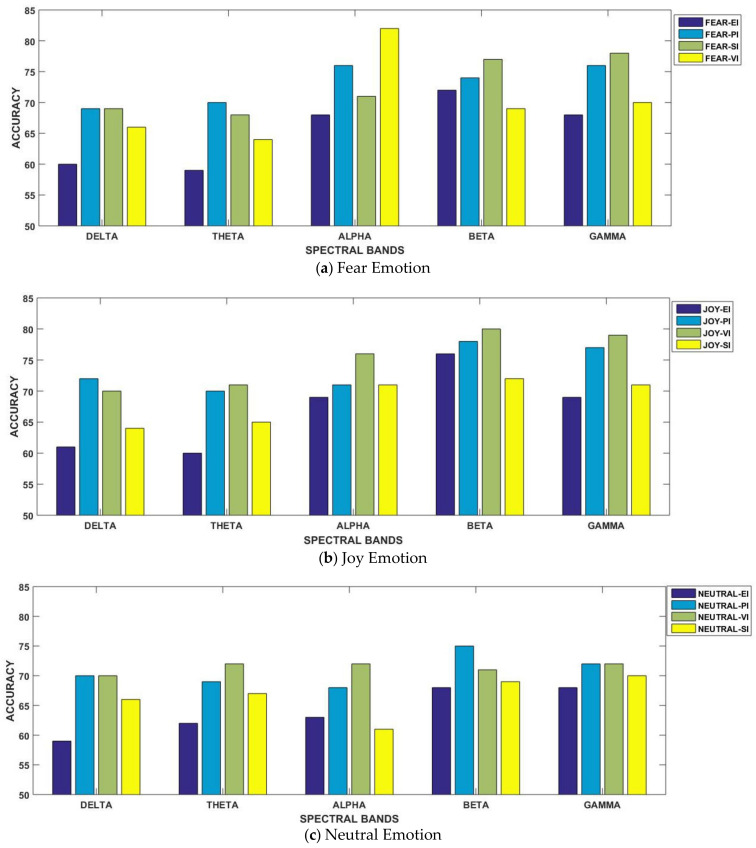
Comparison of classification accuracies amongst all emotion-evoking paradigms for all considered spectral bands.

The results are displayed for all considered frequency bands in Table 3. For better understanding, the results are separately illustrated for each emotional state in Figure 4a–c. In the figure, each graph is presented for each of the three considered emotional states of fear, joy and neutral. Their accuracies with all considered spectral bands are also elaborated for better comparison. From Figure 4a, it can be observed that the fear emotional state was the most accurate in beta and gamma bands for three paradigms. However, in the case of the SI scenario, the best classification performance was achieved in the alpha spectral band. In contrast to fear, the joy emotional state achieved the best performance in emotional imagery and video-induced paradigms within the gamma band. Similarly, the best accuracies were also achieved in higher spectral bands for neutral state in all four paradigms. Specifically, gamma band results showed very minor variations in classification performance for all the four considered paradigms, as mentioned in Figure 4c.

### 4.2. EEG Dynamics/Cortical Activations

**Figure 5:**
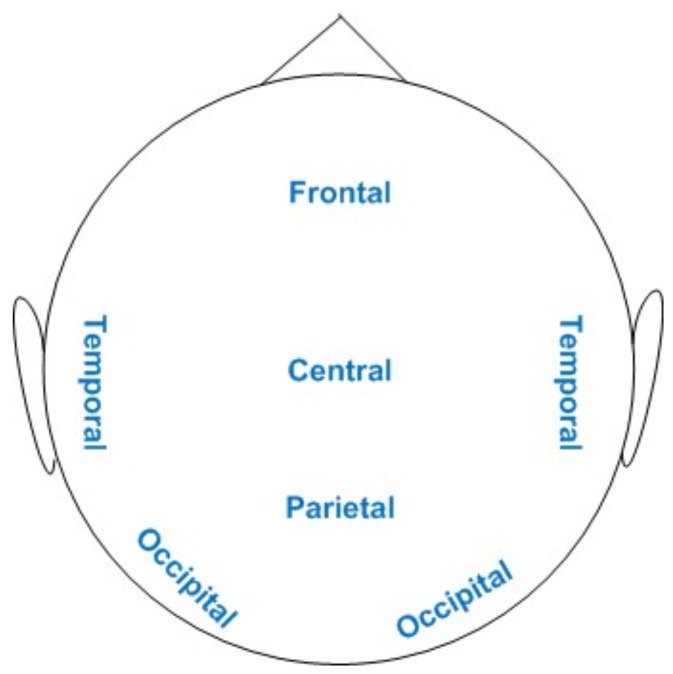
Different locations/regions in the human brain.

As mentioned in the previous section, band power features in terms of spatial filters obtained with CSP algorithms were calculated for each emotional state in each of the four considered paradigms. In order to better understand the EEG cortical activations, orientation markers for different locations of human brain are presented in Figure 5. The figure comprises major brain locations: frontal, temporal, central, parietal and occipital regions.

**Figure 6:**
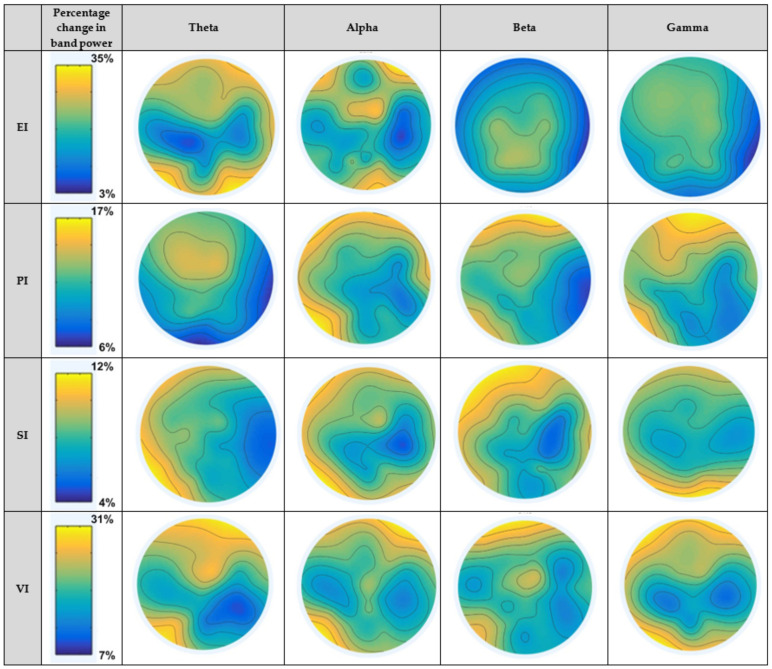
Topographical maps for fear vs. joy emotional states in each of the four considered paradigms for different spectral bands.

Figure 6 depicts the average neural patterns changes for fear vs. joy emotional states in different brain locations (as elaborated in Figure 5). The results demonstrate that neural signatures existed in different spectral bands, keeping in consideration different emotion-evoking paradigms. The first column displays paradigms EI, PI, SI and VI. The second column shows the percentage range of decrease/increase in band power during a test interval during stimulus as compared to a reference interval before stimulus. In the case of the EI paradigm, the change ranged from 3% to 35%. For PI, SI and VI scenarios, the percentage change ranged from 6% to 17%, 4% to 12% and 7% to 31%, respectively. The results obtained in the figure are elaborated as follows:

#### 4.2.1. Within Theta Band

The results of PSD changes in the theta band for the EI paradigm show high cortical activation in the frontal and occipital regions. SI paradigm induced activation in the frontal, temporal, and occipital sites. In the case of the VI paradigm, frontal, temporal, and parietal regions were activated, whereas the EI paradigm showed activation in frontal and occipital areas. While performing statistical analysis, no significant difference was observed among emotional states in theta band for SI and EI paradigms. For the VI paradigm, theta cortical activity showed significant differences at temporal and parietal sites. Considering the picture-induced (PI) paradigm, theta oscillations showed significant differences at frontal and central sites.

#### 4.2.2. Within Alpha Band

The results of cortical dynamics for fear vs. joy emotional states in the alpha band for paradigms under consideration show high activation on different regions, as mentioned in the figures. While performing statistical analysis, alpha oscillations were found to be significantly different in frontal and parietal regions for the SI paradigm. For the VI paradigm, alpha activity was significantly different at the frontal and temporal sites. In the case of the EI paradigm, alpha changes were significant at the frontal region. The PI paradigm showed significant band power changes in alpha oscillations in the frontal and temporal regions.

#### 4.2.3. Within Beta Band

Cortical activations within the beta band for the EI paradigm showed higher activations in the parietal and central regions. For rest of the three paradigms, frontal and temporal regions displayed higher activations. From the statistical analysis, the SI paradigm exhibited significantly higher beta power changes at the frontal and temporal regions. For the VI paradigm, interestingly, beta oscillations showed significant differences at three sites that included the frontal, temporal, and central regions. In the case of the EI paradigm, the beta oscillation changes were significant at the central site. The PI paradigm produced significant differences at the temporal and frontal sites.

#### 4.2.4. Within Gamma Band

In the case of the PI and EI paradigms, frontal and temporal regions produced higher activation. In the SI scenario, occipital and parietal sites were activated. From the statistical analysis, the SI paradigm produced a significant difference for fear vs. joy emotional states at the occipital site. Gamma band power changes were significant in occipital and frontal sites for the VI paradigm. Gamma band produced significant differences at the temporal and frontal sites in the case of the PI paradigm. No significant difference was noted for the EI paradigm.

## 5. Discussion

To achieve the objectives set for the presented research work, we explored the EEG correlates of fear, joy, and neutral human emotional states evoked with four different emotion-inducing paradigms. Classification accuracies were evaluated. Topographical maps were obtained to gain a better understanding of these emotions in different stimuli presentation scenarios. We have performed data analysis by considering data for each stimuli presentation paradigm separately. This considered discrete classification of the samples such that a class was assigned to each sample. For each block of each of the considered emotion-evoking paradigms, features were extracted from the relevant time segment.

### 5.1. Classification Performance Analysis

The results for all considered frequency bands along with emotion-evoking paradigms are presented in Table 3. For better understanding, the results are separately illustrated for each emotional state in Figure 4a–c. We observed that the high-frequency bands of beta and gamma mostly produced better results, independent of the paradigm. Previous neuroscience studies, such as Li and Lu [40], have found that beta and gamma bands of EEG are more relevant for emotion classification. Zhang et al. [41] and Zheng et al. [43] also deduced that higher frequency bands of beta and gamma contributed to human emotional responses rather than lower frequency bands. Our findings are consistent with existing results, but because we studied multiple stimuli-presentation paradigms, deviations were observed. Most previous studies have worked on a single stimulus; therefore, our study may have different contrasting new results. One of the exceptions here is for sound-inducing scenario in which the fear emotional state achieved higher accuracy in the alpha band. Actually, when we reviewed EEG-based emotion recognition studies, variations and deviations in observations and results were found in the neuro-imaging research community as well. As reported by Güntekin et al. [31], some previous studies explored spontaneous EEG modulation of the frequency band (and specifically alpha band) after the application of emotional stimulus. Ljubomir et al. [47], when contrasted with other emotional states, found that an aversive movie clip yielded significant alpha desynchronization. Taniguchi et al. [13] worked on multiple paradigms for emotion elicitation and they found that in the case of auditory stimuli, there is an imbalance between the right and left hemispheres over the central area in the alpha band. Shahabi et al. [44] found better classification accuracies in theta, alpha and beta bands. Eijlers et al. [25] reported a strong classification for happy and disgust emotions in higher frequency ranges. In the case of negative emotions, including sadness and fear, strong differences were found for the alpha frequency band in the centroposterior region. As in our case, we obtained good accuracy for the sound-inducing paradigm within the alpha band for the fear emotional state.

**Figure 7:**
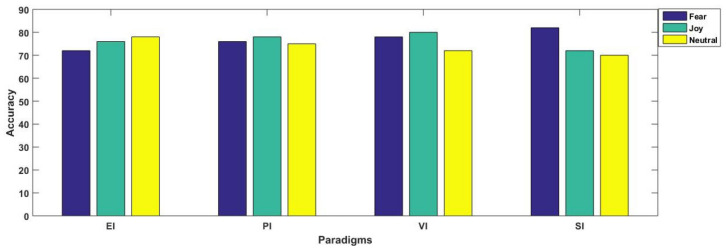
Comparing classification performance for each of the three emotional states in each of the four considered emotion-evoking paradigms.

**Figure 8:**
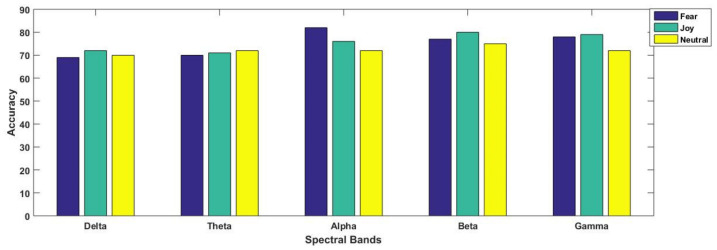
Comparing classification performance achieved for each of the three emotional states in each of the five considered spectral bands.

For better understanding, the results are separately illustrated for each emotional state in Figure 7 and Figure 8. In Figure 7, each graph is presented for each of the three considered emotional states of fear, joy and neutral. Their accuracies with all considered spectral bands are also elaborated for better comparison. From Figure 8, we observe that in three paradigms, fear emotional state achieved the highest accuracy in beta and gamma spectral bands. However, in the case of the SI scenario, the highest accuracy was reported within the alpha band. In contrast to fear, joy showed the best performance in emotional imagery, in picture-and video-induced paradigms, especially in the gamma band. For neutral state, the accuracies are quite comparable amongst different paradigms, although the beta band seemed to provide better performance as compared to gamma in the case of the neutral state. Figure 7 and Figure 8 specifically present results for the highest mean accuracies. In Figure 7, the best mean classification accuracies are compared with respect to emotion-evoking paradigms for each of the three emotional states independent of the spectral band. On the contrary, in Figure 8, the best mean accuracies achieved for each emotion with respect to spectral bands are compared, independent of the emotion-evoking paradigm.

Observing Figure 6 and Figure 7, one can draw a number of observations regarding variations in classification performance with respect not only to emotions, but within spectral bands, and even in paradigms. Here, we find that the highest accuracy was achieved for the joy emotional state in the majority of the paradigms, except sound-induced, in which fear emotion achieved the best performance. Furthermore, Figure 8 depicts that emotion recognition can be best performed while considering higher frequency bands of gamma and beta. In their study, Li et al. [12] classified positive, negative, and neutral emotional states, and concluded that positive emotions are easy to separate compared to other emotional states. They presented their findings through PCA feature visualizations, showing that the dots for positive emotion were more easily separated from the neutral negative emotion dots. From our results as mentioned in Figure 7, we found agreement in all the three paradigms of emotional imagery, picture-and video-induced, whereas there was conflict in the case of the sound-inducing paradigm. Here, instead of joy, fear had a better classification accuracy.

Considering the reviewed literature, it is difficult to compare classification performance because the studies differed in analyzing targeted emotional states, elicitation paradigms, time period to record the activity, features extracted, etc. Just a handful studies have covered emotional imagery. Some of these studies are listed in Table 4. While working on the DEAP dataset, Zhang et al. [41] classified emotional states with support vector machine (SVM) and reported the highest accuracies in beta and gamma spectral bands. Li and Lu [39,40], Jatupaiboon et al. [42] and Zheng et al. [43] have also reported better classification performance in higher spectral bands. In our work, we also obtained the highest mean classification accuracy in the gamma spectral band. Lacoviello et al. [11] recorded EEG data while participants experienced emotional imagery for recalling any unpleasant/disgusting odor. The authors reported accuracy above 90%. Reviewing these studies, it can be determined that accuracies have been reported in different ranges. Some studies have achieved accuracy around 80%, whereas a large body of work reported results between 70 and 75%. In the case of the presented work, because it used different stimuli paradigms, exact comparisons with other relevant studies are not easy or simple. Nevertheless, having a mean accuracy greater than 70% for most of the paradigms represents good, comparable results.

### 5.2. Cortical Dynamics with Respect to Stimulus Paradigms

In this work, band power features in terms of spatial filters obtained with CSP algorithms were calculated for each emotional state in each of the four considered paradigms. Figure 6 depicts the average neural pattern changes for fear vs. joy emotional states. In general, these pictures show that CSP filters appeared as generally smoother and physiologically more relevant, with strong weights in relevant brain regions, as expected from findings in the literature [26,32,33,45,46,65]. With respect to each of the considered paradigm, the findings are elaborated subsequently.

#### 5.2.1. SI Paradigm

In the case of the SI paradigm, no significant difference was observed among emotional states in the theta band. Alpha oscillations were significantly different in the frontal and parietal regions. Significantly higher beta power changes were found for joy infrontal and temporal regions. Gamma oscillations were significantly different for fear and joy emotional states at the occipital site. While reviewing the literature focusing on emotion elicitation with sounds or auditory stimuli, we observed that various features have been explored and studied [26,32,33]. In one study, Lee et al. [26] reported a decrease in alpha power within the right and left frontal brain regions. Moreover, phase lag index values for each of the frequency bands of delta, theta, alpha, beta, and gamma were calculated. The feature showed an increase in negative emotions vs. neutral states. In their study, Holler et al. [32] recorded EEG data while participants listened to their own favorite music. The study reported stronger alpha oscillation responses in occipital and parietal regions. Moreover, beta cortical activity was also reported while listening to music in most of the subjects. Alberto Ara and Marco-Pallares [33] studied frontal theta power synchronization for music-evoked pleasantness. EEG was recorded with 25 participants. Stronger theta oscillation synchronization was reported in temporal and frontal sites with higher degrees of pleasantness.

#### 5.2.2. VI Paradigm

For the VI paradigm, theta cortical activity showed significant difference at the temporal and parietal sites within the theta band. Alpha oscillations were significantly different at the frontal and temporal sites. Interestingly, beta oscillations showed significant differences in the frontal, temporal, and central regions. Gamma band power changes were significant in the occipital and frontal sites. Costa et al. [45] extracted features based on the synchronization index (SI) while recording EEG data for happiness and sadness emotions evoked with audio–visual stimuli. The study reported an increase in SI values, especially in sadness states at the frontal site. Moreover, happiness emotion was strongly synchronized in frontal and occipital regions [45]. Lee et al. [46] conducted a study with forty participants who watched film clips to evoke emotional categories of negative, positive, and neutral conditions. The study analyzed three EEG features based on correlation, coherence, and phase synchronization. Different findings were reported based on the feature under consideration. The statistical analysis for theta oscillation responses produced significantly lower correlation in the frontal region but higher correlation in the temporal and occipital regions for negative emotions. Within the alpha band, a stronger correlation was found for the negative state at occipital and parietal electrode regions. Considering the coherence scenario, higher values were reported for negative emotions within the theta oscillation band [46]. Eijlers et al. [25] reported lower gamma oscillation responses in temporal and frontal regions for happy emotions, whereas a negative state of disgust induced a strong response at the temporal site only. The study recorded EEG data while subjects viewed different film clips to evoke emotions of happiness, fear, sadness, and disgust. In the case of fear, the study found reduced alpha oscillation responses at the central posterior site. Wang et al. [16] analyzed the DEAP dataset [16] for different emotional states using the phase locking value (PLV). The results showed stronger PLVs for negative emotions as compared to positive emotions. Moreover, frontal and temporal sites were found to be more strongly associated with emotional activity as compared to other regions.

#### 5.2.3. EI Paradigm

The EI paradigm showed no significant difference in the case of theta and gamma bands for any region. Alpha changes were significant at the frontal region. In the case of the EI paradigm, beta oscillation changes were significant at the central site within the beta band.

#### 5.2.4. PI Paradigm

Considering the picture-induced (PI) paradigm, theta oscillations showed significant differences at frontal and central sites. Interestingly, significant band power changes were found in alpha, beta and gamma oscillations in the frontal and temporal regions. Schubring and Schupp [53] conducted a study with sixteen participants to analyze EEG-based emotion data while the participants viewed erotic and romantic pictures. The study reported a decrease in alpha and beta oscillation power responses in posterior and anterior sites for erotic images in comparison to romantic images. Miskovic et al. [65] performed experiments with young adult subjects who viewed different pictures varying in valence for pleasant, unpleasant, and neutral scenarios. Stronger coherence was reported for highly arousing pictures at prefrontal and posterior sites within the beta band.

## 6. Highlights and Conclusions

From the conducted study, the results are summarized in Table 5, where ** refers to significant differences (with *p*< 0.05). These findings can be elaborated as follows:

1. With respect to the spectral band, beta and alpha oscillation responses have produced the highest number of significant results considering all the paradigms under consideration. Theta and gamma responses produced significant results, although not greater than alpha and beta;
2. With respect to brain region, the frontal lobe produced the most significant results, irrespective of paradigms and spectral bands. After frontal, the temporal site played an effective role in generating statistically significant findings;
3. With respect to stimulus presentation paradigms, video-based stimuli produced the highest number of statistically significant features. After VI, pictures produced better results as compared to the remaining paradigms of EI and SI. This shows that the visual appearance of a stimulus plays an effective role in emotion recognition;
4. With respect to emotional states, the negative emotion of fear produced stronger band power changes as compared to the joy state, irrespective of spectral band and stimulus presentation paradigm.

Considering classification performance:

1. The positive emotional state of joy was better classified as compared to fear and neutral states in most of the paradigms;
2. Beta and gamma oscillations reported higher accuracies as compared to other spectral bands in most of the paradigms;
3. No specific stimulus could outperform others with respect to classification accuracy.

From the conducted study and comparison with the related literature, we observe that it is quite difficult to identify any single location or spectral band or EEG feature that could produce common findings and observations independent of stimulus presentation paradigms. Cortical activation may vary with respect to the time window under consideration, spectral band, and brain site. The question being addressed in this study could be further explored in detail. Additional positive and negative emotional states, such as anger, disgust, pride, and pleasantness could be explored while considering different stimuli. CSP-based features were extracted and analyzed here for emotion recognition. Other features and techniques, such as deep learning, phase synchronization, and functional connectivity could also be studied. This work provides a good contribution towards designing EEG-based systems for human emotion recognition that could work effectively in different real-time scenarios.

## Figures and Tables

**Table 1:** List of EEG-based studies for emotion recognition identifying synchronization/de-synchronization in different spectral bands.

Study	Emotional States/Stimulus Presentation Paradigms	Synchronization/De-Synchronization	Frequency Band
Knyazev et al. [36]	angry, happiness and neutral conditions	Synchronization	Delta
Alberto Ara and Marco-Pallares [33]	music-evoked pleasantness	Synchronization	Theta
Balconi and Lucchiari [34]	emotional stimuli as compared to neutral one	Synchronization	Theta
Zhang et al. [35]	frightening facial stimuli in comparison to neutral ones	Synchronization	Theta
Lee et al. [26]		De- Synchronization	Alpha
Otten and Jonas [52]	using emotion-evoking words and compared pleasant, unpleasant, and neutral conditions	De- Synchronization	Alpha
Meng et al. [27]	displaying pictures from International Affective Picture System (IAPS) database	De- Synchronization	Alpha
Mennella et al. [28]	displaying pictures from International Affective Picture System (IAPS) database	De- Synchronization	Alpha
Uusberg, A. et al. [29]	-	Synchronization	Alpha
Güntekin et al. [31]	-	Synchronization	Alpha
Schubring and Schupp [53]	erotic and romantic pictures	De- Synchronization	Alpha and Beta
Schutter, Putman, Hermans, and van Honk [38]	angry faces	Synchronization	Beta
Güntekin and Başar [37]	negative emotions in comparison to positive while subjects were viewing IAPS images	Synchronization	Beta
Eijlers et al. [25]	happy emotions	De- Synchronization	Gamma
Sato et al. [54]	negative emotions as compared to neutral and happy states	Synchronization	Gamma
Jung et al. [55]	negative states vs. neutral	Synchronization	Gamma

**Table 2:** Blocks distribution with respect to emotion-inducing paradigm and emotional states.

Paradigms	EI			PI			VI			SI		
Emotion	F	J	N	F	J	N	F	J	N	F	J	N
No. of Trials	10	10	10	45	45	45	10	10	10	45	45	45
Total no. of Trials for each paradigm	30			135			30			135		
Duration of each trial	60 s	60 s	60 s	7 s	7 s	7 s	60–180 s	60–180 s	60–180 s	6–10 s	6–10 s	6–10 s
Time duration considered for each trial	last 55 s	last 55 s	last 55 s	last 5 s	last 5 s	last 5 s	last 55 s	last 55 s	last 55 s	last 5 s	last 5 s	last 5 s
Time window length (s) for each epoch	1 s	1 s	1 s	1 s	1 s	1 s	1 s	1 s	1 s	1 s	1 s	1 s
No. of epochs	550	550	550	225	225	225	550	550	550	225	225	225
Total no. of epochs for each paradigm	1650			675			1650			675		

**Table 3:** Mean classification accuracies for all considered frequency bands in each of the four considered emotion-evoking paradigms.

Paradigm	EI			PI			VI			SI		
Emotional State/Spectral Band	Fear vs. Rest	Happy vs. Rest	Neutral vs. Rest	Fear vs. Rest	Happy vs. Rest	Neutral vs. Rest	Fear vs. Rest	Happy vs. Rest	Neutral vs. Rest	Fear vs. Rest	Happy vs. Rest	Neutral vs. Rest
(1–3 Hz)	60	61	59	69	72	70	69	70	70	66	64	66
(4–7 Hz)	59	60	62	70	70	69	68	71	72	64	65	67
(8–13 Hz)	68	69	63	76	71	68	71	76	72	82	71	61
(14–30 Hz)	72	76	68	74	78	75	77	80	71	69	72	69
(31–50 Hz)	68	69	68	76	77	72	78	79	72	70	71	70

**Table 4:** List of studies using EEG signals to perform emotion recognition.

Study	Type of Study (Emotion Recognition/Others)	Emotion-Inducing Paradigm	Classifier	Classification Performance	Relevant Frequency Band/Brain Regions
Gao et al. [4]	Valence and Arousal	Videos	SVM	62.01%	Gamma frequency band from frontal and temporal regions
Zhuang et al. [8]	Joy, neutrality, sadness, disgust, anger and fear	Self-induced	SVM	54.52%	Higher frequency band/temporal, frontal and occipital sites.
Li et al. [51]	Valence and Arousal	Music	KNN	95.70% 95.69%	Gamma frequency band
Jatupaiboon et al. [42]	Positive vs. negative emotional states	Pictures	SVM	85.41%	Gamma frequency band at frontal region
Zhang et al. [41]	Four emotional states (joy, fear, sadness and relaxation)	Videos	SVM	59.13%	Beta and gamma bands/frontal and parietal sites
Zheng et al. [43]	Positive, neutral and negative	Videos	KNN, SVM, DBNs	83.99%	Beta and gamma bands
Kothe et al. [10]	Positive vs. negative	Self-induced	Logistic Regression	71.3%	-
Lacoviello et al. [11]	Disgust vs. relax	Self-induced	SVM	>90%	-
Li and Lu [39]	Happiness vs. Sadness	Pictures	Linear SVM	93.5%	Gamma band (30–100 Hz)
Author’s work	Fear vs. Rest	Self-induced	LDA	72%	Beta
	Joy vs. Rest			76%	Beta
	Neutral vs. Rest			68%	Beta/gamma
	Fear vs. Rest	Picture-Induced	LDA	76%	Gamma
	Joy vs. Rest			78%	Beta
	Neutral vs. Rest			75%	Beta
	Fear vs. Rest	Video-Induced	LDA	78%	Gamma
	Joy vs. Rest			80%	Beta
	Neutral vs. Rest			72%	Gamma/theta/alpha
	Fear vs. Rest	Sound-Induced	LDA	82%	Alpha
	Joy vs. Rest			72%	Beta
	Neutral vs. Rest			70%	Gamma

**Table 5:** Summary of ANOVA results extracted with respect to stimulus presentation paradigms, spectral bands, and brain regions. Statistical significance (** *p* < 0.05).

	EI					PI					SI					VI				
	F	O	C	Te	Pa	F	O	C	Te	Pa	F	O	C	Te	Pa	F	O	C	Te	Pa
θ						** 0.004		** 0.003											** 0.03	** 0.01
α	** 0.001					** 0.01			** 0.005		** 0.001				** 0.02	** 0.003			** 0.02	
β			** 0.01			** 0.02			** 0.01		** 0.03			**		** 0.004		** 0.001	** 0.04	
γ						** 0.003			** 0.02			** 0.004				** 0.01	** 0.005			

## Data Availability

Data recorded from our experimental work are currently under further analysis; the data will be uploaded when this process is complete.

## Appendix A

Details for stimuli used in the videos-induced paradigm.

**Table A1:** List of videos shown to the participants [48].

S. No.	Videos	Emotional State
1.	*Lights Out* movie trailer	Fear
2.	Best Vacations: Jumping	Joy
3.	Earth/Moon Orbit 3D Animation	Neutral
4.	Video clip from *Insidious* movie	Fear
5.	Caught red-handed	Joy
6.	Solar System Video	Neutral
7.	Conjuring official trailer	Fear
8.	Stunning China—UNESCO World Heritage	Joy
9.	Box Plant Basics—Corrugated Box Basics	Neutral
10.	Die in Disaster Movies	Fear
11.	Tourism Sites in Pakistan	Joy
12.	Planet Earth Rotation 3D	Neutral
13.	Scene from *The Eye*—Horror movie	Fear
14.	Berlin City Tour	Joy
15.	Glow-effect Neon	Neutral
16.	Snake catcher in Indian forest	Fear
17.	BBC nature documentary, 2016	Joy
18.	Earth/Moon Orbit 3D Animation	Neutral
19.	Female Restroom—Horror clip	Fear
20.	Nat Geo Wild HD Ocean of Giants	Joy
21.	Box Plant Basics–Corrugated Box Basics	Neutral
22.	Frightening Creepy Clown	Fear
23.	10-month-old babies	Joy
24.	Solar System Video	Neutral
25.	Scene from *The Conjuring 2*	Fear
26.	Roller Coaster & Candy Coaster	Joy
27.	Planet Earth Rotation 3D	Neutral
28.	Fear of Snakes	Fear
29.	Army Man surprises his 8-year-old daughter	Joy
30.	Box Plant Basics—Corrugated Box Basics	Neutral
